# Supplementary figures and images for: Evolutionary and Gene Expression Analyses Reveal New Insights into the Role of LSU Gene-Family in Plant Responses to Sulfate-Deficiency
Source: Plants (Basel). 2022 Jun 7;11(12):1526. doi: 10.3390/plants11121526 (PMC9229004; doi:10.3390/plants11121526)

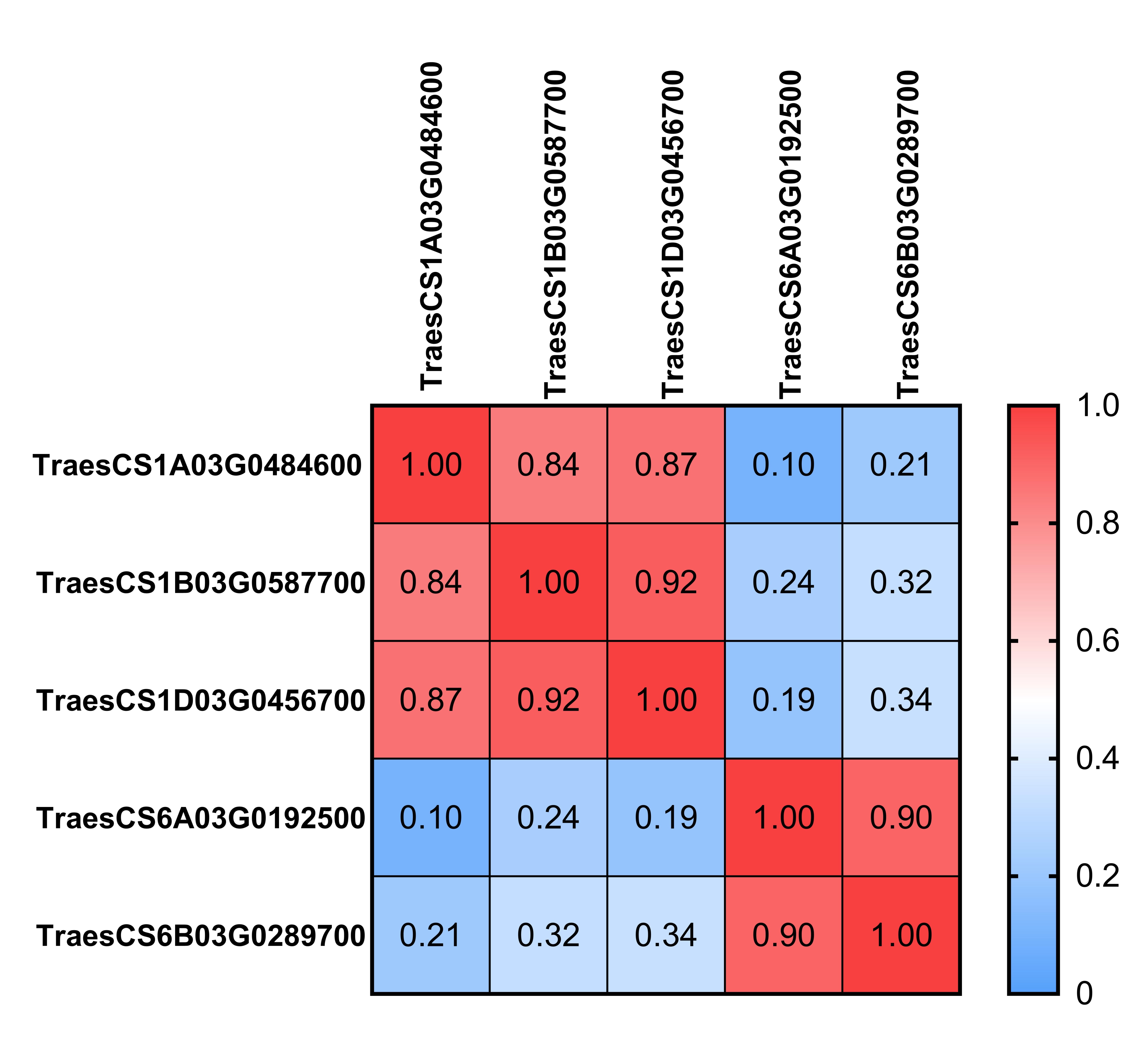

Supplement: Supplementary file 1 [file plants-11-01526-s001.zip › Figure S6.jpg]

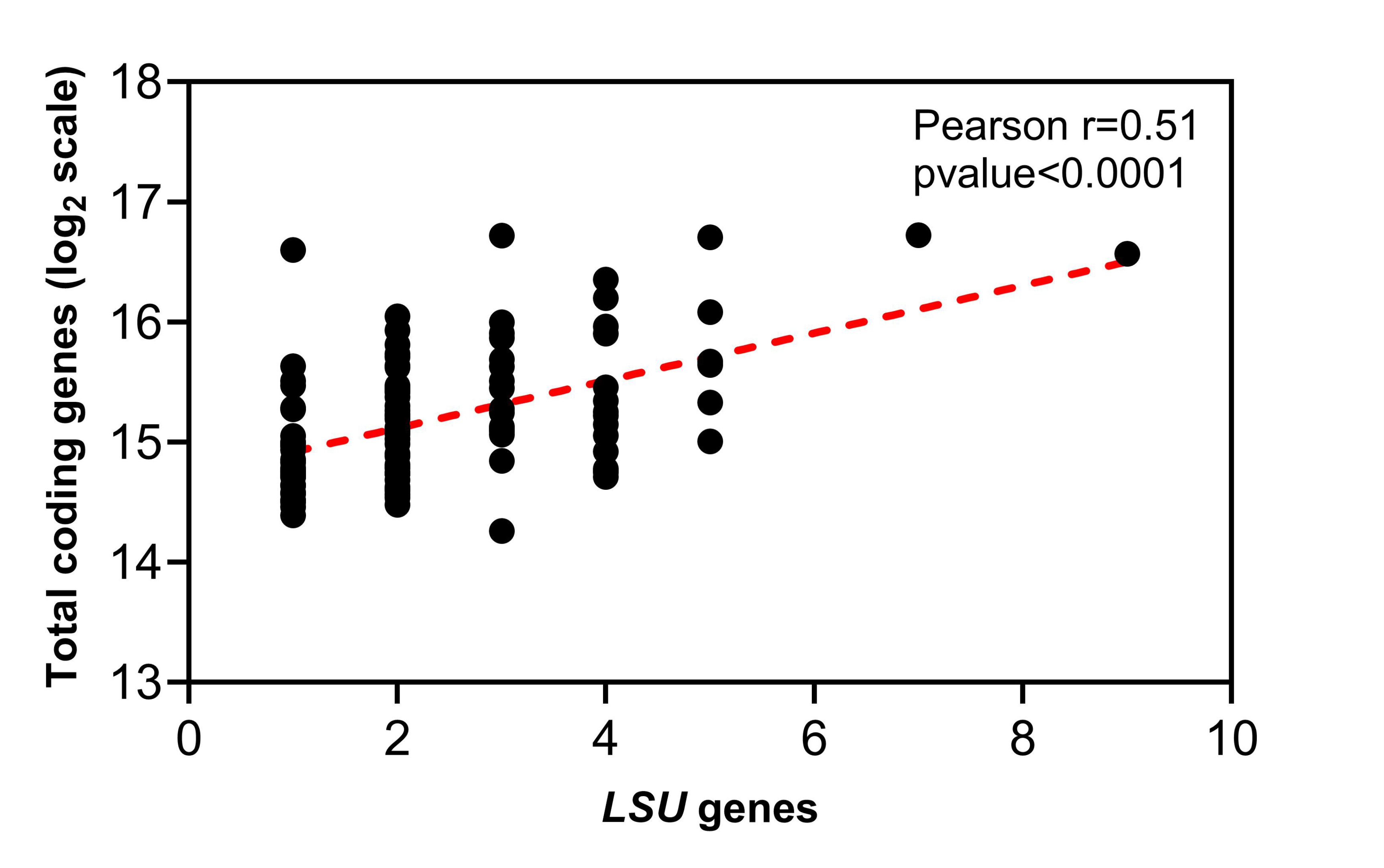

Supplement: Supplementary file 1 [file plants-11-01526-s001.zip › Figure S1.jpg]

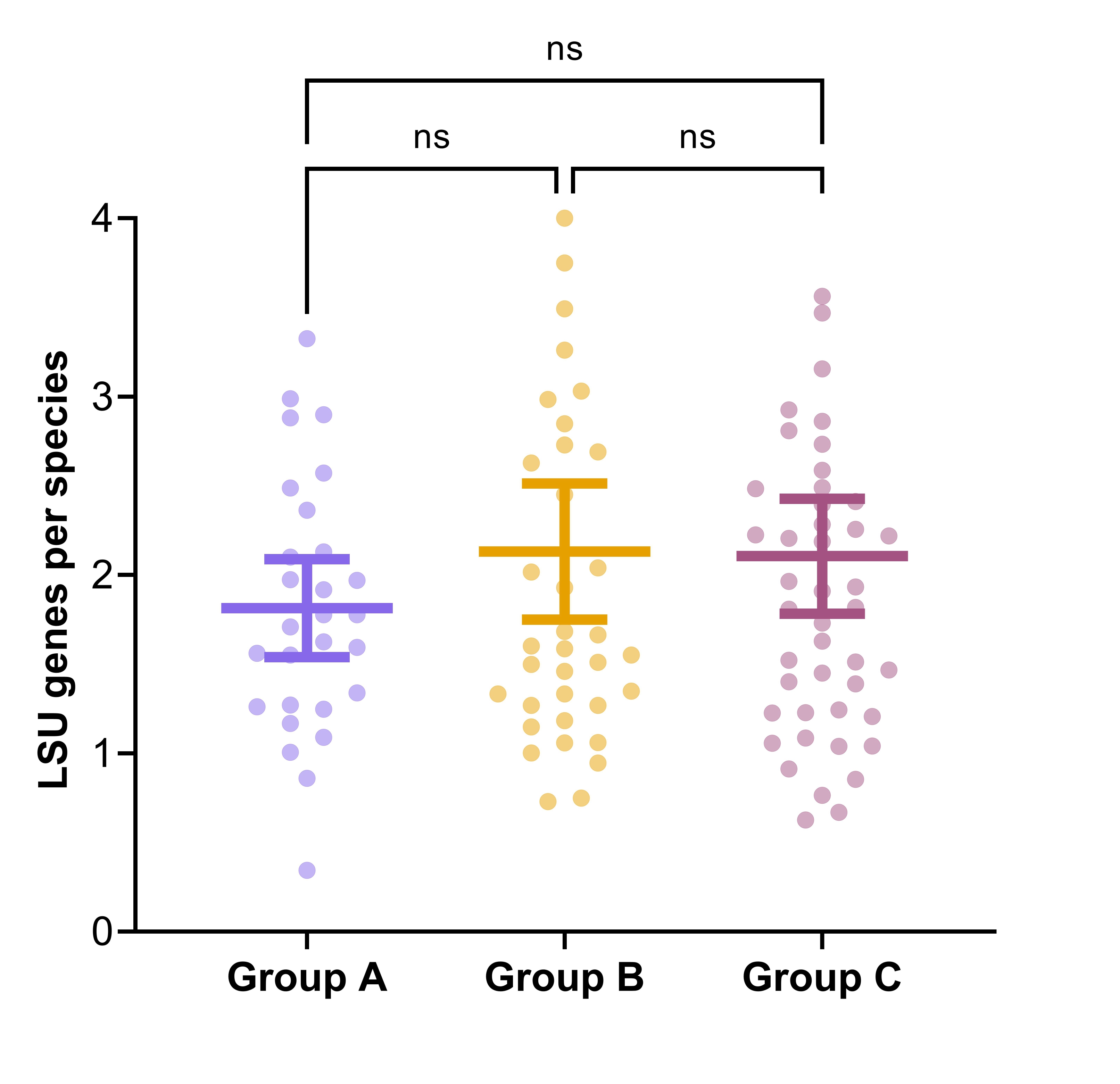

Supplement: Supplementary file 1 [file plants-11-01526-s001.zip › Figure S2.jpg]

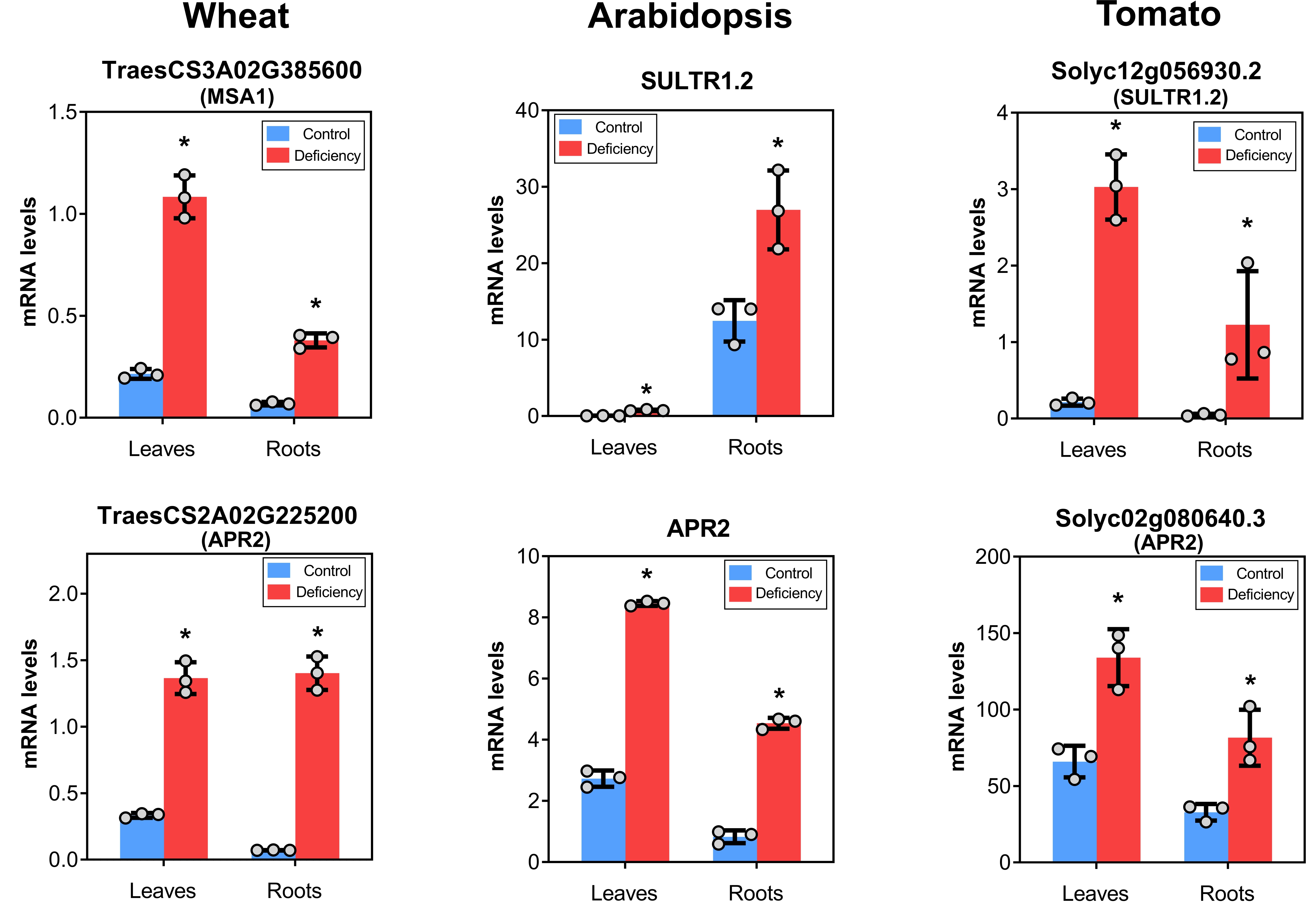

Supplement: Supplementary file 1 [file plants-11-01526-s001.zip › Figure S3.jpg]

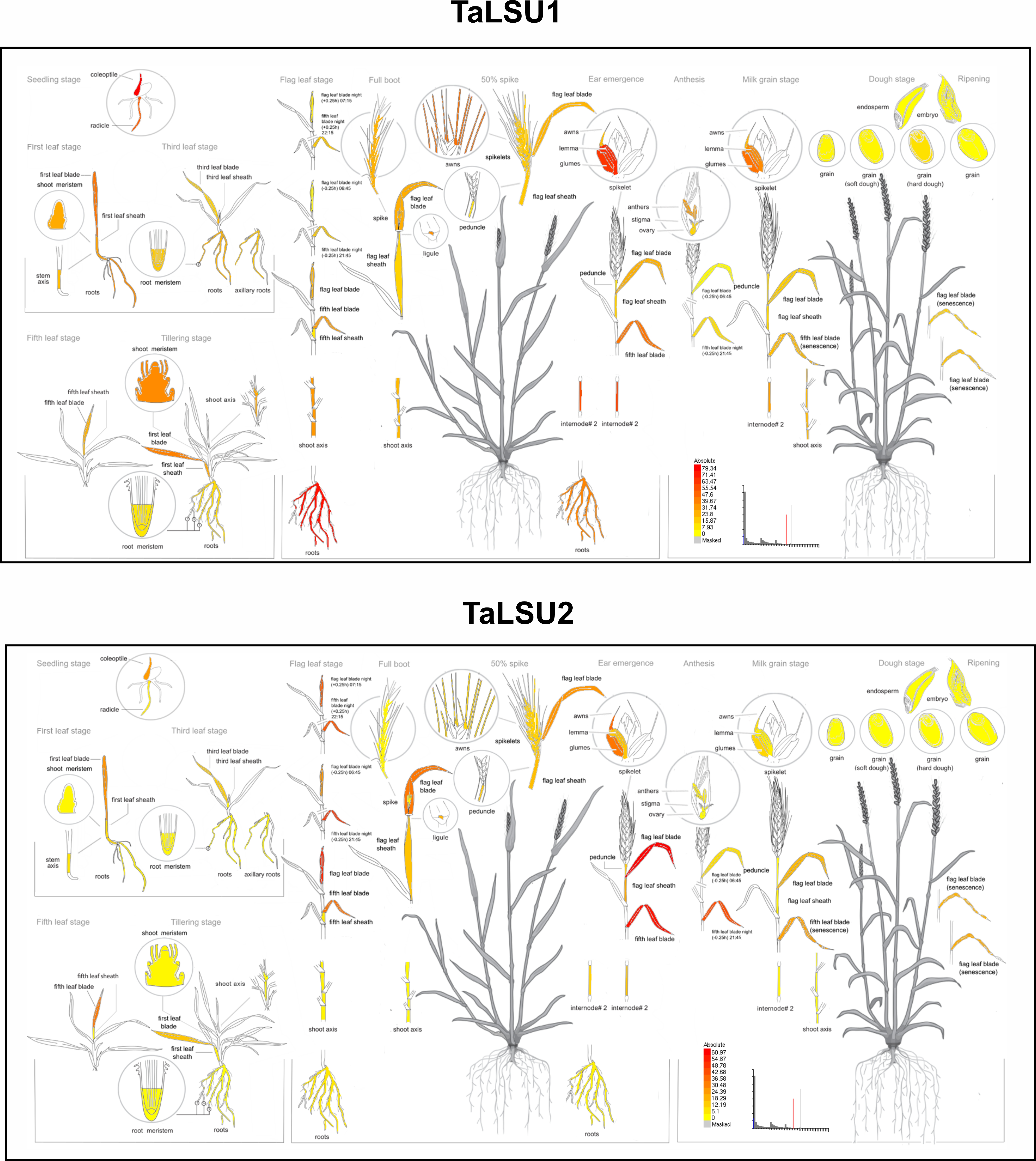

Supplement: Supplementary file 1 [file plants-11-01526-s001.zip › Figure S4.jpg]

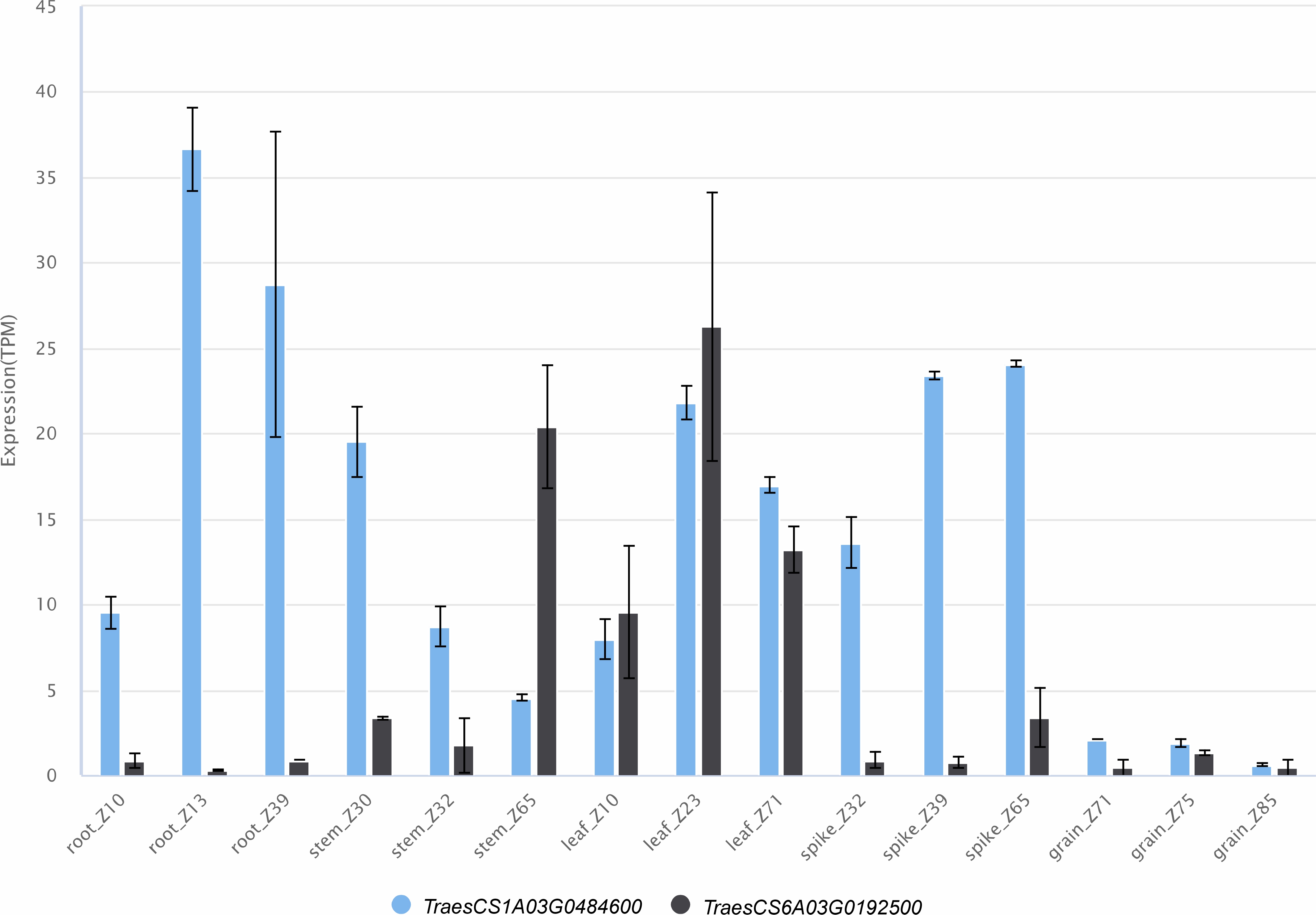

Supplement: Supplementary file 1 [file plants-11-01526-s001.zip › Figure S5.jpg]
